# Complete Genome Sequence of the Marine Carbazole-Degrading Bacterium Erythrobacter sp. Strain KY5

**DOI:** 10.1128/MRA.00935-18

**Published:** 2018-08-30

**Authors:** Felipe Vejarano, Chiho Suzuki-Minakuchi, Yoshiyuki Ohtsubo, Masataka Tsuda, Kazunori Okada, Hideaki Nojiri

**Affiliations:** a Biotechnology Research Center, The University of Tokyo, Tokyo, Japan; b Collaborative Research Institute for Innovative Microbiology, The University of Tokyo, Tokyo, Japan; c Graduate School of Life Sciences, Tohoku University, Sendai, Japan; University of Delaware

## Abstract

We determined the complete genome sequence of Erythrobacter sp. strain KY5, a bacterium isolated from Tokyo Bay and capable of degrading carbazole. The genome consists of a 3.3-Mb circular chromosome that carries the gene clusters involved in carbazole degradation and biosynthesis of the photosynthetic apparatus of aerobic anoxygenic phototrophic bacteria.

## Abstract (precis)

We determined the complete genome sequence of Erythrobacter sp. strain KY5, a bacterium isolated from Tokyo Bay and capable of degrading carbazole.

## ANNOUNCEMENT

In recent years, bacterial strains capable of degrading carbazole, a carcinogenic and mutagenic nitrogen-containing aromatic contaminant in fossil fuels (1–3), have been isolated from marine environments (4–8). While the nucleotide sequences of the carbazole-degradative *car* gene clusters have been determined, no complete genome sequences of these marine isolates have been obtained to date. Several Erythrobacter strains belong to marine aerobic anoxygenic phototrophic (AAP) bacteria, a ubiquitous group of proteobacteria that predominantly inhabit the euphotic zone in the oceans and are thought to contribute greatly to the carbon cycle in those habitats (9, 10). Recent reports of sequenced Erythrobacter sp. genomes have mainly provided information regarding their genome structure and the characteristics of their photosynthetic gene cluster (PGC) (11–16), while information about the presence or organization of xenobiotic catabolic genes is still scarce (12, 16), even though the capability to degrade such compounds has been reported in a few strains (5, 17). Here, we report the complete genome sequence of Erythrobacter sp. strain KY5, a Gram-negative AAP bacterium isolated from Tokyo Bay and capable of degrading carbazole.

Erythrobacter sp. KY5 was isolated from a mixed culture obtained from Tokyo Bay water samples, enriched in the presence of carbazole (0.5% [wt/vol]) in vitamin-supplemented (18), filter-sterilized seawater medium containing KH_2_PO_4_, yeast extract (10 mg/liter), and Fe-EDTA (3 mg/liter) (carbazole-containing seawater [CAR-SEA] medium). After carbazole depletion was observed, strain KY5 was isolated by spreading 100-μl aliquots of the culture onto 8.5-cm plates containing the same enrichment medium solidified with 16 g/liter bacteriological agar. Degradation of the compound was evidenced by the formation of clear zones around the colonies and later confirmed upon culturing the strain in CAR-SEA medium.

Cultivation of strain KY5 in CAR-SEA medium at 30°C for 4 days was followed by DNA extraction using the Wizard genomic DNA purification kit (Promega) per the manufacturer’s instructions. Whole-genome sequencing was performed using Illumina PCR-free and mate pair libraries in a MiSeq sequencer. Genome assembly was done using Newbler (454 Life Sciences) and finished using AceFileViewer (www.ige.tohoku.ac.jp/joho/gf/AceFileViewer.php) and GenoFinisher (http://www.ige.tohoku.ac.jp/joho/gf_e/GenoFinisher.php) for gap closing. Gene prediction and functional annotation were first done using the NCBI Prokaryotic Genome Annotation Pipeline (19) and the Microbial Genome Annotation Pipeline (20). Annotation results of the two pipelines were compared and checked for inconsistencies using GenomeMatcher (21), producing a curated annotation file that combined the results of both annotation pipelines.

The genome of strain KY5 consists of one 3,311,272-bp circular chromosome with an average G+C content of 60.77%. Functional annotation revealed a total of 3,080 coding sequences, 43 tRNA genes, and one copy of the rRNA genes. A 10.5-kbp *car* gene cluster was found with the configuration *carDAaCBaBbFE*, followed by a gene encoding a putative reductase of the initial carbazole degradation enzyme, carbazole 1,9a-dioxygenase, whose terminal oxygenase component is encoded by the *carAa* gene (22). Notably, 2.5 kbp upstream of the *car* gene cluster is an anthranilate-coenzyme A ligase ortholog, suggesting that this strain degrades carbazole through a coenzyme A intermediate and not catechol, which is consistent with the absence of anthranilate or catechol oxidoreductase genes. Strain KY5 also carries the conserved 39-kbp-long Sphingomonadales clade type III PGC (23) with genes for the biosynthesis of the photosynthetic apparatus components.

### Data availability.

The genome sequence of Erythrobacter sp. strain KY5 has been deposited in DDBJ/ENA/GenBank under the accession number CP021912.
